# Newborn Genetic Screening Improves the Screening Efficiency for Congenital Hypothyroidism: A Prospective Multicenter Study in China

**DOI:** 10.3390/ijns10040078

**Published:** 2024-11-29

**Authors:** Liang Ye, Yinhong Zhang, Jizhen Feng, Cidan Huang, Xiaohua Wang, Lianshu Han, Yonglan Huang, Hui Zou, Baosheng Zhu, Jingkun Miao

**Affiliations:** 1 Department of Pediatrics, Chongqing Health Center for Women and Children, Women and Children’s Hospital of Chongqing Medical University, Chongqing 401147, China; yeallen2020@163.com; 2 Department of Medical Genetics, NHC Key Laboratory of Preconception Health in Western China, The First People’s Hospital of Yunnan Province, Affiliated Hospital of Kunming University of Science and Technology, Kunming 650100, China; zyh8920002@163.com; 3 Department of Genetic, Shijiazhuang Maternal and Child Health Hospital, Shijiazhuang 050006, China; 18731160921@163.com; 4 Neonatal Disease Screening Center, Hainan Women and Children’s Medical Center, Haikou 570206, China; 13807652278@139.com; 5 Department of Genetics, Inner Mongolia Maternity and Child Health Care Hospital, Hohhot 750306, China; wangxiaohua2222@163.com; 6 Department of Pediatric Endocrinology and Genetic Metabolism, Shanghai Institute for Pediatric Research, Xinhua Hospital, Shanghai Jiaotong University School of Medicine, Shanghai 200092, China; hanlianshu@xinhuamed.com.cn; 7 Department of Guangzhou Newborn Screening Center, Guangzhou Women and Children’s Medical Center, Guangzhou Medical University, Guangdong Provincial Clinical Research Center for Child Health, Guangzhou 510623, China; xxhuang321@163.com; 8 Neonatal Disease Screening Center, Jinan Maternity and Child Health Hospital Affiliated to Shandong First Medical University, Jinan 250001, China; zouhui819@163.com

**Keywords:** congenital hypothyroidism, newborn screening, genetic screening, *DUOX2*

## Abstract

Newborn congenital hypothyroidism (CH) screening has been widely used worldwide. The objective of this study was to evaluate the effectiveness of applying biochemical and gene panel sequencing as screening tests for CH and to analyze the mutation spectrum of CH in China. Newborns were prospectively recruited from eight hospitals in China between February and December 2021. Clinical characteristics were collected. Second-generation sequencing was used to detect four CH-related genes, and the genetic patterns of the pathogenic genes were analyzed. We analyzed the relationship between genotype and biochemical phenotype. A total of 29,601 newborns were screened for CH. Gene panel sequencing identified 18 patients, including 10 patients affected by biochemically and genetically screened disorders and 8 patients affected by solely genetically screened disorders. The predictive positive value of genetic screening was 34.62%, which was much greater than that of biochemical screening alone (17.99%). A total of 94 cases of congenital thyroid dysfunction were confirmed by biochemical and genetic screening, including 30 CHs and 64 isolated hyperthyrotropinemia (HTT), with an incidence of 1/987 for CH and 1/463 for HTT, and a total incidence of 1/315 for hypothyroidism. The incidence rate and number of patients in Jinan were the highest, and the incidence rates in Shijiazhuang and Shanghai were the lowest. The gene mutation rate in this study was 19.1%, mainly *DUOX2* mutation. The most common variant of *DUOX2* was c.1588A>T(p.Lys530*). There was only a difference in sFT4 between groups with gene mutations and those without mutations. Genetic screening is a supplement to biochemical screening. Combining biochemical screening with genetic screening is useful for improving screening efficiency. The incidence of CH in China according to a multicenter study of nearly 30,000 NBS surveys was 1/315. *DUOX2* gene mutations are commonly detected in these patients.

## 1. Introduction

Congenital hypothyroidism (CH) is one of the most common neonatal endocrine and metabolic diseases and affects approximately 1:2000–1:4000 infants worldwide [1]. According to newborn screening data, the incidence rate of CH in China is higher than the global average level [2,3]. CH can lead to growth retardation and permanent intellectual disability [4]. However, early screening and treatment could significantly improve adverse outcomes. Thus, most countries conduct newborn screening (NBS) for CH, which is widely used as the third preventive intervention for birth defects [5,6].

Traditional biochemical screening for thyroid stimulating hormone (TSH) from the dried blood spot (DBS) is used as the mainstream method of CH screening at this stage [7]. However, TSH levels are affected by many factors, such as gestational age, birth weight, feeding, and basic diseases [8,9,10]. With increasing research on the pathogenesis of CH, the genetic origin of its pathogenesis has gradually been recognized [11,12]. According to the latest report, newborn genetic screening has been proven to be successful when single monogenetic disease or targeted genetic sequencing panels are used [13,14]. However, no study has evaluated the effectiveness of combined biochemical screening and genetic screening for CH in a large population. Moreover, the incidence of CH NBS in the Chinese population has been reported, but there is a lack of joint screening studies for multiple genes and related research on CH-related genes and clinical phenotypes [15].

This study employed targeted genetic sequencing to detect four candidate genes related to CH and combined it with biochemical screening to determine the latest incidence of CH in China. Furthermore, the study analysed the relationship between the genotype and clinical phenotype of children with CH and the mutation spectrum of CH pathogenic genes in selected Chinese populations. In addition, the study laid a theoretical foundation for neonatal screening and clinical diagnosis of CH and future gene therapy.

## 2. Materials and Methods

### 2.1. Study Population and Design

From 21 February 2021 to 30 December 2021, a total of 29,601 newborn infants who participated in the newborn screening program and gene screening project were recruited for this study. Eight hospitals participated in the multicenter project, including all the subjects who received CH screening via DBS collection. Each participating unit should include appropriate subjects. Leaflets of the free gene screening program for newborns should be distributed among the guardians of the subjects. They should be informed and should sign an informed consent to confirm the inclusion of the subjects. The study design and protocol were reviewed and approved by the Ethics Committee of Xinhua Hospital Affiliated with Shanghai Jiaotong University School of Medicine. The eight hospitals were selected to represent the nationwide population, as they are regional tertiary hospitals located in East China, West China, South China, and North China. The participating hospitals were Xinhua Hospital affiliated with Shanghai Jiaotong University School of Medicine, Guangzhou Women and Children’s Medical Center, Jinan Maternity and Child Care Hospital, Shijiazhuang Maternal and Child Health Care Hospital, Chongqing Health Center for Women and Children, the First People’s Hospital of Yunnan Province, Inner Mongolia Maternity and Child Health Care Hospital, and Hainan Women and Children’s Medical Center.

### 2.2. Newborn Screening

The screening, diagnosis, treatment, and reevaluation of CH were in line with the criteria of the “Consensus statement on the diagnosis and management of congenital hypothyroidism” in China [16]. Briefly, for 7 days after 72 h from birth, the newborns were exclusively breastfed, and blood was collected from the heel and dripped on special filter paper (Whatman903, Liding, Guizhou, China) to form DBSs. A time-resolved fluoroimmunoassay (Perkin-Elmer, Waltham, MA, USA) was used to measure the TSH level. The cutoff value of TSH varies according to the different screening methods used at each hospital (see Supplementary Table S1 for specific values). If the TSH level elevated the upper limit of normal, the infants would be recalled, heel blood would be collected for a second time and the TSH level would be retested. If the new TSH value was still above the upper limit of normal, the infants would be recalled again, and their venous blood would be collected to measure the levels of serum TSH and FT4.

### 2.3. Diagnosis of CH

**Figure 1:**
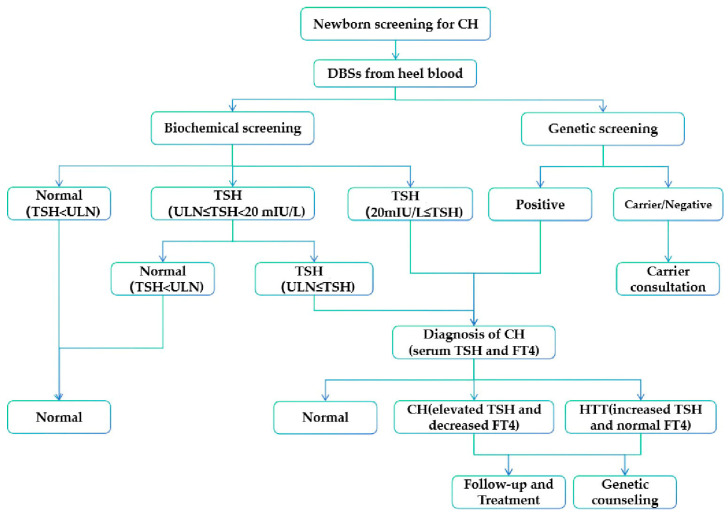
The screening and diagnosis process of CH. CH, congenital hypothyroidism; HTT, isolated hyperthyrotropinemia; ULN, upper limit of normal value; TSH, thyroid stimulating hormone.

TSH Reagent Kit (Abbott, IL, USA) and Free T4 Reagent Kit (Abbott, IL, USA) were used to measure Serum TSH and FT4 levels. Serum TSH and FT4 levels were determined via an electrochemiluminescence immunoassay (ECLIA) assay, ARCHITECT i system, and Alinity i analysis System (Abbott, IL, USA). The diagnosis of CH is based on elevated TSH levels and decreased FT4 levels. Isolated hyperthyrotropinemia (HTT) is characterized by increased TSH and normal FT4. The cutoff values of serum TSH and FT4 vary according to the different screening methods used at each hospital (Supplementary Table S1 for specific values). Thyroid ultrasonography was performed to evaluate thyroid development. The information regarding the diagnosed children, including birth time, sex, birth weight, gestational week, and family history of thyroid disease, was collected and recorded via the neonatal disease screening registration form. The screening procedure is shown in Figure 1.

### 2.4. Genomic DNA Extraction and Sequencing

Genomic DNA was extracted from the DBS using a genomic DNA extraction system kit (QIAamp DNA Blood Midi Kit, Qiagen, Hilden, Germany). The DNA concentration was 3–25 ng/μL, and the DNA purity (OD 260/280) reached 1.8–2.0. The genomic DNA was broken into small DNA fragments with a main band of 100–500 bp by a Covaris LE220 ultrasonic instrument (MA, USA), and then the broken DNA fragments were screened by magnetic beads. Library construction, end repair, and 3′-end tailing were performed using a fragmentation module (Vazyme, Nanjing, China). The four CH-related genes *DUOX2* (NM 014080), *DUOXA2* (NM 207581), *TSHR* (NM 000369), and *PROP1* (NM 006261) were selected via a gene capture strategy via the Agilent 2100 Bioanalyzer (Beijing, China) and BMG following the manufacturer’s protocol. High-throughput sequencing of the qualified enriched libraries was performed on a MEGISEQ-2000 sequencer (BGI, Shenzhen, China).

### 2.5. Analysis of Mutation Data

The sequencing results were analyzed via bioinformatics methods. Spilt, comparison, and quality control were performed on the original sequencing data. We performed a search of internal databases, dbSNP, ESP6500, gnomAD, and 1000 Genomes. Prediction software was then used to predict whether the mutations were conserved and the contribution of the mutations to CH pathogenesis. We searched the HGMD, PubMed, Clinvar, and other databases and literature related to variation, and variants were analyzed following the basic criteria from the American College of Medical Genetics (ACMG) guidelines [17,18].

### 2.6. Treatment and Follow-Up

After diagnosis, all the children were given L-T4 (levothyroxine sodium) therapy, their thyroid function was assessed after one month of treatment, and the dosage was adjusted according to the test results of thyroid function, height, weight, and individual differences. Under the condition of normal thyroid function, the patient was rechecked at 2–3 months of age and once every 3–4 months of age from 1 to 3 years of age, and physical and intellectual development was evaluated regularly.

### 2.7. Statistical Analysis

Statistical analysis was performed via SPSS (version 25.0, NY, IBM Corp.). The chi-squared test was used to compare counting data between groups. A test of normal distribution and homogeneity of variance was used for measurement data. Data that were not normally distributed are expressed as medians (M), 25th percentiles (P25), and 75th percentiles (P75). Normally distributed data are expressed as the means and standard deviations. The difference between each group was tested by a non-parametric rank sum test. *p* < 0.05 was considered to indicate a statistically significant difference.

## 3. Results

### 3.1. Newborn Screening for CH

In this study, a total of 29,601 newborns underwent CH screening. A total of 94 cases of congenital thyroid dysfunction were confirmed, among which 46 were male infants and 48 were female infants. The incidence rate of hypothyroidism was 1/315. According to the clinical phenotype, there were 30 CHs and 64 HTTs in 94 patients. The gestational ages were mostly between 37 and 42 weeks, and only four infants (4%) were preterm. Birth weights were normal in 89 cases, low in 2 infants (2%), and macrosomic in 3 newborns (3%). The median blood spot TSH levels of the 94 patients was 11.52 mIU/L (P25–P75: 9.03–16.93 mIU/L). After serological examination, the median serum TSH level of CH infants was 20.28 μIU/mL (P25–P75: 12.11–49.80 μIU/mL), and that of FT4 was 13.65 pmol/L (P25–P75: 9.55–15.38 pmol/L). Among them, the incidence rate and number of patients in Jinan were the highest, followed by those in Chongqing and Yunnan, and the incidence rates in Shijiazhuang and Shanghai were the lowest (Table 1 and Table 2).

### 3.2. Comparison of Biochemical Screening and Genetic Screening Results

In total, 499 infants had positive traditional biochemical results, and the positive rate of initial screening was 1.68% (499/29,601). A total of 478 (95.79%) cases were successfully recalled and underwent serological confirmation testing. According to the serological confirmation test, 86 infants (43 males and 43 females) were diagnosed with hypothyroidism. The positive predictive value of biochemical screening was 17.99% (86/478). The recorded demographic data and clinical features of the patients are presented in Supplementary Tables S2 and S3. CH-related genes were detected by targeted next-generation sequencing (NGS) in 29,601 newborns. In total, 62 strains tested positive via genetic screening, and the positive rate of genetic screening was 0.21% (62/29,601). A total of 59 patients (95.2%) were successfully recalled and underwent a serological confirmation test. According to the serological confirmation test, 18 infants were diagnosed with hypothyroidism, including 6 with CH and 12 with HTT. The positive predictive value of genetic screening was 34.62% (18/59), which was greater than that of biochemical screening. The above results suggest that genetic screening improves the detection rate of hypothyroidism.

We then compared positive results from traditional biochemical screening and genetic screening. According to the results of the combined genetic screening and traditional biochemical screening, 94 children were diagnosed. Among them, 76 were positive in biochemical screening and negative in genetic screening, 10 were positive in both types of screening, and 8 were negative in biochemical screening and positive in genetic screening. The above results indicate that 8 out of every 94 patients were missed by traditional biochemical screening; fortunately, they could be identified by genetic screening. Genetic screening plays an important role in improving the efficiency of CH screening and could be used as a supplement to biochemical screening. Compared with those of combined screening, the sensitivities of traditional biochemical screening and genetic screening were 91.49% and 19.15%, respectively. Combined screening improves the sensitivity of screening (Table 3).

### 3.3. Mutation Patterns of CH-Related Genes

**Figure 2:**
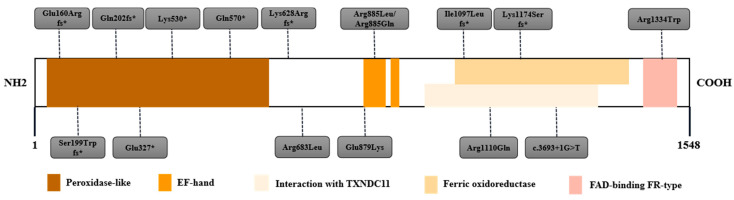
Mutation sites in the secondary structure of *DUOX2*. Sixteen mutation sites distributed in the secondary structure of *DUOX2* protein. *DUOX2* contains 5 functional domains, peroxidase-like domain, EF-hand domain, Interaction with TXNDC11 domain, ferric oxidoreductase domain, and FAD-binding FR-type domain.

On the basis of our literature review, we designed a targeted sequencing panel that included four causative genes: *DUOX2*, *DUOXA2*, *TSHR*, and *PROP1*. Because of the critical role of the above four genes in thyroid hormone synthesis and action, and the strong correlation between their mutations and CH, they were included in the gene sequencing panel for CH. Among the 29,601 newborns, 62 tested positive for the *DUOX2* gene, which is related to thyroid dyshormonogenesis. Eighteen patients with pathogenic variants in the *DUOX2* gene were eventually diagnosed with hypothyroidism. As shown in Table 4, the most common variant of *DUOX2* was c.1588A>T(p.Lys530*) with a frequency of 33.33%, followed by c.3329G>A(p.Arg1110Gln) (11.11%). Among the 16 mutation sites of *DUOX2*, 6 were located in the peroxidase-like domain, 4 in the ferric oxidoreductase domain, 3 in the EF-hand domain, and 1 in the FAD-binding FR-type domain, all of which play key roles in the function of *DUOX2*. Only the c.1883delA(p.Lys628Argfs*) and c.2048G>T(p.Arg683Leu) were located in the nonfunctional domain (Figure 2). No mutations in *TSHR*, *DUOXA2*, or *PROP1* were detected.

### 3.4. Relationships Between Genotype and Phenotype

**Figure 3:**
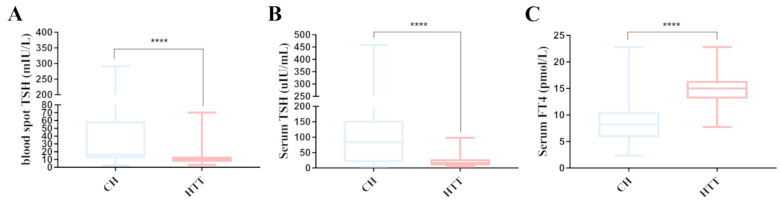
Comparison of serum levels of screening TSH, diagnostic TSH and FT4 among different groups, classified according to clinical phenotype. (**A**) Comparison of TSH levels in dry blood spots between patients with CH and HTT. (**B**) Comparison of TSH levels in serum between patients with CH and HTT. (**C**) Comparison of FT4 levels in serum between patients with CH and HTT. CH, Congenital hypothyroidism; HTT, isolated hyperthyrotropinemia; **** p  <  0.0001.

Ninety-four children were divided into two groups, CH and HTT, on the basis of their clinical phenotype. The biochemical indices of thyroid function were compared between the two groups (Figure 3). The results revealed that the dry blood spots and serum TSH levels of patients with CH were significantly greater than those of patients with HTT (*p* < 0.0001). The median sTSH concentration at diagnosis was greater in patients with CH than in those with HTT (*p* < 0.0001). The median FT4 concentration at diagnosis was greater in patients with HTT than in those with CH (*p* < 0.0001).

**Figure 4:**
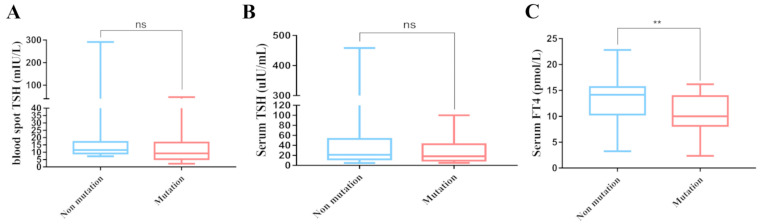
Comparison of serum levels of screening TSH, diagnostic TSH and FT4 among different groups, classified according to whether there is a gene variation. (**A**) Comparison of TSH levels in dry blood spots between patients with no mutation, and mutation. (**B**) Comparison of TSH levels in serum between patients with no mutation, and mutation. (**C**) Comparison of FT4 levels in serum between patients with no mutation, and mutation. ns, *p*  >  0.05; ** *p*  <  0.01.

According to the results of genetic screening, the studied patients were classified into two groups, and the biochemical indices of thyroid function were compared between the two groups (mutation group and no mutation group). The results revealed that only the serum FT4 levels of patients with mutations at diagnosis were significantly lower than those of patients without gene mutations (*p* < 0.01) (Figure 4). There was no significant difference in the levels of blood spot TSH and sTSH between the mutation and non-mutation groups.

Next, we investigated the relationship between the genotype and clinical phenotype of CH patients. Because many types of gene mutations are detected, the c.1588A>T and c.3329G>A mutations and the mutation types with the highest mutation frequency are taken as genotypes. We studied the relationship between the genotypes of high-frequency gene mutation sites and the clinical phenotypes of CH patients (Table 5 and Table 6) and found that the c.1588A>T and c.3329G>A variants were not related to clinical type (CH or HTT). In addition, significant differences in sFT4 levels were detected between patients with no c.1588A>T variant, the c.1588A>T homozygous variant, and the c.1588A>T compound heterozygous variant, indicating that patients with the c.1588A>T variant are associated with low sFT4 levels.

## 4. Discussion

CH is an endocrine disease caused by insufficient synthesis and secretion of thyroid hormones. It is one of the major targets of newborn screening in the world. This study presented nearly 30,000 NBS data points, indicating that the average incidence rate of hypothyroidism (including CH and HTT) in multiple regions was 1/315, which is far higher than the reported incidence rate [1,2,3]. Among them, the incidence rate of CH was 1/897, and the incidence rate of HTT was 1/467. Studies have suggested that the increasing trend of CH incidence might be associated with a reduction in TSH cutoff levels, an increase in the survival of preterm infants, and changes in population demographics [19,20,21]. An increased proportion of CH patients with eutopic glands may also account for this trend [2]. Furthermore, the variability of serum reference ranges (RRs) and the absence of specific neonatal RRs may contribute to an increased likelihood of CH or HTT being misdiagnosed. The question of whether infants with normally positioned glands and mild HTT should receive treatment remains unresolved. Some studies have indicated that it is safe to not treat infants with mild HTT without significant developmental deficits, while others have supported early treatment as a means of preventing potential long-term cognitive problems. Consequently, a follow-up plan must be tailored to the specific circumstances of the child. It is also essential to have a clear understanding of how to manage the dose and course of LT4 [22,23].

Our data revealed that the incidence rate and the number of patients in Jinan were the highest, followed by those in Chongqing and Yunnan and that the incidence rates in Shijiazhuang and Shanghai were the lowest. This finding is consistent with the regional differences in the incidence of CH [21,24]. This may be related to the iodine distribution in the area and the residents’ eating habits. Additionally, differences in CH incidence across geographic regions may also be related to the distribution of populations with CH susceptibility genes. The genetic pathogenesis of CH in most newborns is still unclear, and some cases are related to genetic mutations [25], which are often undetectable by NBS.

NBS for CH is performed routinely in most regions of China, where it has led to the near elimination of intellectual disability caused by this common condition. However, NBS for CH is associated with a high rate of false-negative results, which appears to be inevitable because the TSH and FT4 concentrations at birth are easily affected by maternal and other factors. These factors include premature birth, low birth weight, and central hypothyroidism [9,10]. Furthermore, false positives can cause newborns and their families to be recalled to the hospital for reexamination, which may take a long time. Additionally, newborns at risk of CH may not produce enough TSH in the first few weeks after birth, and the screening results may be false negative at this time. The guidelines recommend that for twins, a second screening and follow-up should be performed 2 weeks after birth or 2 weeks after the first screening. However, it is important to note that not all subsequent cases of hypothyroidism are cases of CH that have been overlooked. It is therefore essential to perform a thorough differential diagnosis. In our study, genetic screening was performed on 29,601 neonates via targeted next-generation sequencing, and 8 false-negative CH cases were identified, which translates into 8 out of every 94 newborns benefiting from the implementation of CH genetic screening. Following the incorporation of the aforementioned eight children into the follow-up treatment system, it was observed that they exhibited normal thyroid development, thyroid function, neuropsychiatric development, and growth and development. The above results and those of previous studies confirmed the effectiveness of genetic screening, which could be used as a supplement to biochemical screening [26,27]. Therefore, NBS for CH faces challenges, and combining biochemical screening with genetic testing is crucial to improve screening sensitivity.

CH is mainly caused by the underdevelopment of the thyroid gland, dyshormonogenesis, and central congenital hypothyroidism. Therefore, we selected the hotspot mutant genes *DUOX2* and *DUOXA2* in CH in the Chinese population, which are associated with disorders of thyroid hormone synthesis and secretion. The TSHR gene, which is associated with thyroid dysplasia, was also selected; TSH resistance caused by loss-of-function mutations in *TSHR* is the most common genetic factor leading to congenital hypothyroidism. In addition, central congenital hypothyroidism is strongly associated with variants in the *PROP1* gene, which is usually associated with pituitary hormone underproduction. In particular, combined pituitary hormone deficiency caused by mutations in the *PROP1* gene is associated with decreased TSH secretion [28]. Although CH screening is primarily aimed at primary hypothyroidism, testing for *PROP1* gene mutations may reveal some undetected cases of secondary hypothyroidism [29]. The inclusion of the *PROP1* gene in the range of tests is aimed at further research into potential genetic factors. Although this is not the main purpose of the screening, it undoubtedly provides extremely valuable genetic information. With respect to the relationship between genotype and biochemical phenotype, significant differences were observed in sFT4 levels between patients with and without gene variants and, compared with the patients in the no mutation group, most patients with gene variants had lower FT4 levels. *DUOX2* is involved mainly in the production of peroxide protein complexes and catalyzes the synthesis of thyroid hormones in thyroid follicular cells [30]. The protein encoded by *DUOX2* is involved in the iodization process of thyroxine synthesis, which is closely related to the generation of FT4 [31]. In this study, all genes were merged with one or two *DUOX2* mutations. These children exhibit various clinical manifestations, including CH and HTT, indicating that mutations in *DUOX2* are not necessarily related to clinical classification [31]. Our research revealed that homozygous mutations or compound heterozygous mutations of c.1588A>T and c.3329G>A lead to abnormal thyroid function, but the c.1588A>T and c.3329G>A variants had no correlation with clinical classification (CH or HTT). In addition, the c.1588A>T variant was related to sFT4 levels, indicating that patients with the c.1588A>T variant have more severe hypothyroidism. The damage to the functional domain caused by the c.1588A>T variant is related to peroxidase-like activity [32,33]. The region where c. 1588A>T is located in the main functional domain for *DUOX2* to exert peroxidase activity. The above results indicate that *DUOX2* mutations in major structural domains, such as the peroxidase-like region, have a significant effect on thyroid function. Studies have shown that mutations in *DUOX2* can initiate CH, but the clinical phenotype, primarily the manifestation of transient congenital hypothyroidism, is variable [34,35]. Our study had a short follow-up period, and the clinical phenotypic data were not comprehensive. Variable phenotypes are presumed to be caused by other undetected genetic mutations, individual differences, stochastic phenomena, or environmental factors [32,36]. With regards to *DUOX2* over 100 different mutations have been reported but there is ambiguity about the number of these which are truly pathogenic. Therefore, pathogenic gene screening for patients with CH, enrichment of the mutation spectrum, and identification of the relationships between gene mutations and phenotypes are the recommended steps to achieve accurate clinical diagnosis and treatment of CH.

Although we performed relatively systematic and comprehensive screening for candidate genes associated with CH, some limitations should be considered when reviewing our findings. First, this is a highly selected population, and a larger sample size study is needed to further confirm the correlation between gene mutations and clinical phenotypes; future investigations should expand the scope of genetic testing to include a larger number of genes associated with hypothyroidism. Second, most of the patients had a short follow-up period, and the clinical phenotypic data were not comprehensive. Therefore, it is not analytically conducive to determine the correlation between the detected mutation and the clinical phenotype. Finally, it is recommended that the variants identified in this study be subjected to in vitro functional studies. The subsequent studies will perform and analyze three-dimensional models of the missense variants identified in the *DUOX2* gene, with a particular focus on one of the most prevalent variants, p.Arg1110Gln, among others. Therefore, further studies are needed to expand the mutation spectrum of CH and to verify the functions of the associated mutations, which may provide more profound insight into the etiology of CH.

This study reveals that the latest incidence rate of hypothyroidism in China is 1/315 (including CH and HTT) by using routine screening and gene screening methods and provides a basis for research on neonatal CH genetic screening in China. Genetic screening is a supplement to biochemical screening. The incorporation of gene screening and traditional biochemical screening into neonatal CH screening is conducive to avoiding missed diagnoses. The advantages of this study include that it is the first relatively large multicenter study on the genetic diagnosis of children with CH in China, with a comprehensive clinical process for analysis through genetic diagnosis. However, the strategy of CH gene screening is still in the exploration stage, and the genes and loci covered by CH gene screening in Chinese newborns need to be further evaluated. Moreover, our first systematic and comprehensive screening of four CH-candidate genes in Chinese newborns preliminarily proved the necessity, feasibility, and importance of clinical gene screening for neonates and revealed that the main mutated gene of the four genes tested in this study was *DUOX2*.

## Figures and Tables

**Table 1:** Multicenter screening for congenital hypothyroidism.

Region	Screening Number	CH	HTT	Total	Incidence Rate
Shanghai	4888	1	3	4	1/1222
Jinan	4797	7	40	47	1/102
Guangzhou	4813	7	3	10	1/481
Hainan	977	1	1	2	1/489
Chongqing	2988	10	7	17	1/176
Yunnan	3006	3	8	11	1/273
Shijiazhuang	4899	1	0	1	1/4899
Inner Mongolia	3233	0	2	2	1/1616
Total	29,601	30	64	94	1/315

**Table 2:** Clinical features of the 94 cases.

Project	Cases (%)	Project	Cases (%)
Gender		Clinical phenotype	
Male	46 (49%)	CH	30 (32%)
Female	48 (51%)	HTT	64 (68%)
Gestational age (week)		blood spot TSH (mIU/L)	
<37	4 (4%)	<10	35 (37%)
37–42	90 (96%)	10–40	48 (51%)
≥42	0	≥40	11 (12%)
Birth weight (g)		Serum TSH (μIU/mL)	
<2500	2 (2%)	5–20	47 (50%)
2500–4000	89 (95%)	20–100	33 (35%)
≥4000	3 (3%)	≥100	14 (15%)
Gene mutation		Serum FT4 (pmol/mL)	
NO variants	76 (81%)	<6	7 (7%)
*DUOX2* variants	18 (19%)	6–12 ≥12	33 (35%) 54 (58%)

**Table 3:** Diagnostic results.

		Combined Screening		Predictive Value	Total
		+	−		
Biochemical screening	+	86	0	PPV: 100%	86
	−	8	29,507	NPV: 99.97%	29,515
		Sensitivity: 91.4%	Specificity: 100%		
Genetic screening	+	18	0	PPV: 100%	18
	−	76	29,507	NPV: 99.74%	29,583
		Sensitivity: 19.14%	Specificity: 100%		
Total		94	29,504		29,601

+, positive screening; −, negative screening; PPV, Positive Predictive Value; NPV, Negative Predicticv Value.

**Table 4:** Potential pathological variants detected in the present study.

Gene	Position	cDNA Change	Amino Acids Change	ACMG Classification	Mutation Type	No. of Cases	Frequency (%)
*DUOX2*	Exon14	c.1588A>T	p.Lys530*	P	nonsense	12	33.33%
*DUOX2*	Exon25	c.3329G>A	p.Arg1110Gln	P	missense	4	11.11%
*DUOX2*	Exon10	c.2635G>A	p.Glu879Lys	P	missense	3	8.33%
*DUOX2*	Exon20	c.2654G>T	p.Arg885Leu	LP	missense	3	8.33%
*DUOX2*	Exon16	c.1883delA	p.Lys628Argfs*	P	frameshift	2	5.55%
*DUOX2*	IVS28	c.3693+1G>T	/	LP	splicing	2	5.55%
*DUOX2*	Exon5	c.477delC	p.Glu160Argfs*	LP	frameshift	1	2.78%
*DUOX2*	Exon6	c.596delC	p.Ser199Trpfs*	LP	frameshift	1	2.78%
*DUOX2*	Exon6	c.605_621delAGCTGGCGTCGGGGCCC	p.Gln202fs*	LP	frameshift	1	2.78%
*DUOX2*	Exon9	c.978_979delGGinsTT	p.Glu327*	LP	frameshift	1	2.78%
*DUOX2*	Exon15	c.1708C>T	p.Gln570*	LP	nonsense	1	2.78%
*DUOX2*	Exon17	c.2048G>T	p.Arg683Leu	LP	missense	1	2.78%
*DUOX2*	Exon20	c.2654G>A	p.Arg885Gln	P	missense	1	2.78%
*DUOX2*	Exon25	c.3285_3286delTT	p.Ile1097Leufs*	LP	frameshift	1	2.78%
*DUOX2*	Exon27	c.3516_3531delGTCCAAGCTTCCCCAG	p.Lys1174Serfs*	P	frameshift	1	2.78%
*DUOX2*	Exon30	c.4000C>T	p.Arg1334Trp	LP	missense	1	2.78%
Total						36	100%

P, pathogenic; LP, likely pathogenic.

**Table 5:** Relationship between the genotype of c.1588A>T variant and the biochemical results of thyroid function.

Groups	Cases	CH (*n*)	HTT (*n*)	Blood Spot TSH (mIU/L)	Serum TSH (uIU/mL)	Serum FT4 (pmol/L)
No c.1588A>T variant	85	26	59	11.29 (9.01, 16.72)	20.99 (11.68, 45.02)	14.05 (10.27, 16.29)
c.1588A>T homozygous variant	3	2	1	13.84 (12.47, -)	18.41 (17.07, -)	9.62 (5.62, -)
c.1588A>T compound heterozygous variant	6	4	2	16.38 (5.66, 31.14)	24.12 (11.67, 72.64)	9.07 (6.42, 11.50)
Statistics		Χ^2^ = 4.718; *p* = 0.095		H = 0.759; *p* = 0.684	H = 0.278; *p* = 0.870	H = 8.592; *p* = 0.014 *

CH, Congenital hypothyroidism; HTT, isolated hyperthyrotropinemia. * *p* < 0.05.

**Table 6:** Relationship between the genotype of c.3329G>A variant and the biochemical results of thyroid function.

Groups	Cases	CH (*n*)	HTT (*n*)	Blood Spot TSH (mIU/L)	Serum TSH (uIU/mL)	Serum FT4 (pmol/L)
No c.3329G>A variant	91	29	62	11.60 (9.03, 16.77)	20.99 (12.23, 54.28)	13.65 (9.35, 15.43)
c.3329G>A homozygous variant	1	0	1	9.30 (9.30, 9.30)	17.92 (17.92, 17.92)	12.79 (12.79, 12.79)
c.3329G>A compound heterozygous variant	2	1	1	7.70 (6.32, -)	21.27 (9.88, -)	11.17 (10.72, -)
Statistics		Χ^2^ = 0.770; *p* = 0.680		H = 3.003; *p* = 0.223	H = 0.215.; *p* = 0.898	H = 0.947; *p* = 0.623

CH, Congenital hypothyroidism; HTT, isolated hyperthyrotropinemia.

## Data Availability

Access to the data included in the analyses can be provided upon request to the corresponding author.

## Supplementary Materials

The following supporting information can be downloaded at: https://www.mdpi.com/article/10.3390/ijns10040078/s1, Table S1: Normal cut-off value range of thyroid function index; Table S2: Clinical Information, and detected variants of studied patients with CH. Table S3: Clinical Information, and detected variants of studied patients with HTT.
