# Supplementary material for: Newborn Genetic Screening Improves the Screening Efficiency for Congenital Hypothyroidism: A Prospective Multicenter Study in China
Source: Int J Neonatal Screen. 2024 Nov 29;10(4):78. doi: 10.3390/ijns10040078 (PMC11678242; doi:10.3390/ijns10040078)
Supplement: Supplementary file 1 [file IJNS-10-00078-s001.zip › IJNS-3190875-supplementary.pdf]

Supplemental File

Table S1. Normal cut-off value range of thyroid function index.

Region	Project		
	**bsTSH (mIU/L)**	**sTSH(μIU/ml)**	**sFT4(pmol/L)**
Shanghai	<10.00	0.55-4.78	11.50-22.70
Jinan	<8.00	0.34-5.60	9.32-19.64
Guangzhou	<10.00	0.47-10.00	12.00-29.34
Hainan	<7.00	0.27-4.20	12.00-22.00
Chongqing	<8.00	0.35-4.94	9.10-19.24
Yunnan	<8.00	0.30-4.60	10.44-24.38
Shijiazhuang	<7.95	0.35-4.94	9.00-19.04
Inner Mongolia	<9.00	0.27-4.20	12.00-22.00

bsTSH, blood spot TSH; sTSH, serum TSH; sFT4, serum FT4; sFT3, serum FT3.

Table S2. Clinical Information, and detected variants of studied patients with CH.

Patients ID	Gender	Gestational age (week+ day)	Birth weight(g)	blood spot TSH （mU/L）	Serum TSH (uIU/mL)	Serum FT4 (pmol/L)	Variants	Clinical phenotype
1	Male	38	3150	101.00	150.10	3.25	/	CH
2	Female	39	3000	47.47	100.10	2.37	*DUOX2*: c.1883delA(p.Lys628 Argfs*11)/c.1588A>T (p.Lys530*)	CH
3	Male	39+1	3630	15.84	137.80	9.18	/	CH
4	Female	40+3	2550	109.5	458.4	8.01	/	CH
5	Female	38+2	2820	20.44	78.40	9.30	/	CH
6	Female	38+1	3070	8.53	259.00	7.26	/	CH
7	Male	38+4	3750	57.68	327.60	7.87	/	CH
8	Female	38	3210	14.92	63.76	8.14	/	CH
9	Female	40	2760	63.92	141.68	11.64	/	CH
10	Male	40	3260	57.88	150.10	14.41	/	CH
11	Male	38	2830	33.05	150.10	10.83	/	CH
12	Male	40	3250	24.24	66.37	8.95	/	CH
13	Male	40	4160	22.27	100.20	10.80	/	CH
14	Male	40	3250	21.60	4.85	22.82	/	CH
15	Male	40	3250	12.19	150.10	6.38	/	CH
16	Male	40	3400	7.26	23.73	11.20	/	CH
17	Male	39	3250	13.84	100.10	5.62	*DUOX2*: c.1588A>T(p.Lys530 *)/c.1588A>T(p.Lys5 30*)	CH
18	Female	40	3630	14.46	17.07	9.62	*DUOX2*: c.1588A>T(p.Lys530 *)/c.1588A>T(p.Lys5 30*)	CH
19	Female	39	3190	2.21	31.20	3.76	*DUOX2*: c.3285_3286delTT(p.I le1097Leufs*24)/c.26 54G>T(p.Arg885Leu)	CH

20	Male	39	3130	16.77	35.20	8.29	*DUOX2*: c.3516_3531delGTCCAAGCTTCCCCAG(p.Lys1174Serfs*12)/c.2635G>A(p.Glu879Lys)	CH
21	Female	39	3240	13.90	13.15	6.19	/	CH
22	Female	39	3160	14.21	12.70	7.89	/	CH
23	Male	38	3720	11.29	11.15	5.44	/	CH
24	Female	41	3250	59.95	88.67	6.16	/	CH
25	Male	39	3330	14.12	100.10	5.51	/	CH
26	Male	39	3030	13.10	48.31	8.48	/	CH
27	Female	40+2	2700	9.07	32.60	9.62	*DUOX2*: c.3329G>A(p.p.Arg1110Gln)/c.1588A>T(p.Lys530*)	CH
28	Male	38	2760	9.78	5.00	10.34	/	CH
29	Male	40	3460	69.00	5.00	10.34	/	CH
30	Female	38	3250	291.17	1501.10	5.05	/	CH

CH, congenital hypothyroidism.

Table S3. Clinical Information, and detected variants of studied patients with HTT.

Patients ID	Gender	Gestational age (week+ day)	Birth weight(g)	blood spot TSH （mU/L）	Serum TSH (uIU/mL)	Serum FT4 (pmol/L)	Variants	Clinical phenotype
31	Female	39+1	3430	12.60	14.10	21.72	/	HTT
32	Male	37+5	3050	5.35	8.83	14.62	*DUOX2*: c.3693+1G>T/c.477delC(p.GLu160Argfs*16)	HTT
33	Female	38+2	3250	3.15	6.25	15.43	*DUOX2*: c.1883delA(p.Lys628Argfs*11)/c.1708C>T (p.Gln570*)	HTT
34	Male	40+6	3350	23.68	63.49	15.68	*DUOX2*: c.1588A>T(p.Lys530*)/c.2635G>A(p.Glu879Lys)	HTT
35	Male	39	3250	10.41	15.77	18.35	/	HTT
36	Female	35	2300	53.77	12.30	15.29	/	HTT
37	Female	36+1	3160	10.22	12.76	14.37	/	HTT
38	Male	37+1	2260	8.78	37.78	18.64	/	HTT
39	Female	40	3400	11.61	14.13	15.27	/	HTT
40	Male	40+4	2750	10.75	11.23	14.05	/	HTT
41	Female	39+4	3230	11.33	21.12	15.60	/	HTT
42	Male	40+3	3250	9.21	20.99	16.40	/	HTT
43	Male	40+6	3700	13.26	54.28	12.31	/	HTT
44	Female	40+1	2680	8.01	76.89	18.18	/	HTT
45	Male	38+6	2700	9.12	22.62	14.66	/	HTT
46	Female	38+4	3100	19.91	28.03	13.65	/	HTT
47	Female	40+4	3010	15.36	27.51	17.05	/	HTT
48	Female	38	3215	10.00	22.90	14.62	/	HTT
49	Male	39+2	3200	11.75	24.67	16.44	/	HTT
50	Female	40+6	3530	8.99	13.06	10.62	/	HTT

51	Female	39	3050	10.02	6.05	10.65	/	HTT
52	Female	40+2	2720	9.03	16.02	15.64	/	HTT
53	Female	40+4	3450	17.40	98.15	9.35	/	HTT
54	Female	40+1	3140	9.49	19.24	13.66	/	HTT
55	Female	37+6	3400	8.14	17.99	15.33	/	HTT
56	Female	37+3	2950	11.93	37.01	15.24	/	HTT
57	Female	40+2	3050	70.19	30.71	15.31	/	HTT
58	Female	36+2	3550	8.01	23.24	15.32	/	HTT
59	Male	38	3450	8.66	10.56	16.32	/	HTT
60	Male	40+5	3600	12.48	11.63	15.21	/	HTT
61	Female	39+5	2630	8.01	11.33	15.85	/	HTT
62	Female	40+6	3250	9.76	5.65	19.65	/	HTT
63	Male	40	3500	8.01	12.69	16.25	/	HTT
64	Male	38+5	3850	9.55	11.73	16.36	/	HTT
65	Female	39+5	3930	16.02	13.91	15.66	/	HTT
66	Female	39+3	3980	8.22	19.00	15.33	/	HTT
67	Male	38+6	3800	9.06	11.49	14.26	/	HTT
68	Female	39	3180	8.01	23.03	13.65	/	HTT
69	Male	41	3400	8.69	41.57	11.36	/	HTT
70	Female	41+5	3100	8.01	24.54	13.65	/	HTT
71	Male	37	2650	10.29	33.16	11.03	/	HTT
72	Male	40+3	3150	10.08	15.41	15.36	/	HTT
73	Female	40+2	3000	9.03	19.40	13.45	/	HTT
74	Female	40	2740	5.80	7.78	16.19	*DUOX2*: c.2654G>T(p.Arg885Leu)/c.978_979delGGinsTT(p.Glu327Phe1548del)	HTT

75	Male	39	3250	23.03	19.57	18.49	/	HTT
76	Male	40	3230	11.16	26.21	22.81	/	HTT
77	Male	40	2900	10.66	12.23	20.75	/	HTT
78	Male	39	3530	12.47	18.41	9.85	*DUOX2*: c.1588A>T(p.Lys530*)/c.1588A>T(p.Lys530*)	HTT
79	Male	39	4290	5.18	5.02	14.12	*DUOX2*: c.596delC(p.Ser199Trpfs*122)/c.2048G>T(p.Arg683Leu)	HTT
80	Male	40	3030	16.56	26.21	10.19	*DUOX2*: c.2654G>T(p.Arg885Leu)/c.2654G>T(p.Arg885Gln)	HTT
81	Female	37	3900	6.32	9.88	10.72	*DUOX2*: c.1588A>T(p.Lys530*)/c.3329G>A(p.Arg1110Gln)	HTT
82	Female	39	3550	9.30	17.92	12.79	*DUOX2*: c.3329G>A(p.p.Arg1110Gln)/c.3329G>A(p.p.Arg1110Gln)	HTT
83	Female	39	3260	12.98	25.36	9.64	/	HTT
84	Male	34	2620	11.43	6.69	13.26	/	HTT
85	Male	40+3	3650	25.70	12.26	7.77	*DUOX2*: c.1588A>T(p.Lys530*)/c.605_621delAGCTGGCGTCGGGGCCC(p.Gln202 fs*93)	HTT
86	Female	39+5	3250	3.68	15.63	8.52	*DUOX2*: c.3693+1G>T/c.1588A>T(p.Lys530*)	HTT
87	Male	39+5	3250	7.99	92.78	10.34	*DUOX2*: c.4000C>T(p.Arg1334Trp)/c.2635G>A(p.Glu879Lys)	HTT
88	Male	37	2500	11.90	4.80	15.00	/	HTT
89	Male	40	3400	11.60	5.00	15.00	/	HTT

90	Female	40+2	3450	8.14	5.00	15.00	/	HTT
91	Male	39	3100	12.70	9.68	12.45	/	HTT
92	Female	40+6	3500	9.32	7.80	13.40	/	HTT
93	Female	41+2	3540	19.20	41.72	19.64	/	HTT
94	Female	39	4190	9.88	14.94	21.28	/	HTT

HTT, isolated hyperthyrotropinemia.
